# A fusion sparse learning algorithm for fault identification of rolling bearings

**DOI:** 10.1371/journal.pone.0339859

**Published:** 2026-01-05

**Authors:** Yefeng Liu, Jingjing Liu, Yanwei Ma, Shuai Wang, Qichun Zhang

**Affiliations:** 1 Liaoning Key Laboratory of Information Physics Fusion and Intelligent Manufacturing for CNC Machine, Shenyang Institute of Technology, Fushun, Liaoning, China; 2 School of Automation and Electrical Engineering, Linyi University, Linyi, Shandong, China; 3 Department of Basic Courses, Shenyang Institute of Technology, Fushun, Liaoning, China; 4 School of Mechanical Engineering and Automation, Shenyang Institute of Technology, Fushun, Liaoning, China; 5 School of Automation and Electrical Engineering, Shenyang Ligong University, Shenyang, Liaoning, China; 6 School of Creative and Digital Industries, Buckinghamshire New University, High Wycombe, United Kingdom; Donghua University, CHINA

## Abstract

A key part of CNC machine tools is the rolling bearing, and thus, it is vital to employ a data-driven approach for fault diagnosis. This paper proposes a two-stage fusion sparse learning algorithm for fault data processing that can identify and diagnose the fault types of rolling bearings based on sensor measurement data. During the feature extraction phase, temporal features of sequential data within the big data are extracted using a Long Short - Term Memory (LSTM) network. Moreover, the classification learning stage contains a new sparse learning algorithm, which applies $L_{1/2}$ regularization on stochastic configuration networks (SCN). The iterative learning formula combines the alternating direction method of multipliers (ADMM) with the analysis of the quadratic equations theory. Simultaneously, the model’s inequality supervision mechanism is updated based on convergence analysis. This developed algorithm incorporates the benefits of LSTM in extracting temporal data characteristics, along with the sparsity, ease of convergence, and lightweight nature of SCN. Consequently, it mitigates the shortcomings of deep models in end-to-end applications, particularly in terms of interpretability and structural redundancy, thus making it suitable for deployment on edge devices. Finally, a fusion sparse learning model (LSTM-$L_{1/2}$-SCN) is introduced based on the two-stage learning algorithm for rolling bearing fault diagnosis. In the experiments on the benchmark dataset, the optimal sparsity degree of this algorithm for the Sparse Coding Network (SCN) reached 76.66%, which was 30% higher than that of the Pooling-based Sparse Coding Network (PSCN). Moreover, in the experiments based on the dataset of Case Western Reserve University (CWRU), the optimal test classification accuracy achieved was 97.51%, and the optimal sparsity degree for SCN reached 29.39%. These results verify that the proposed algorithm exhibits sparsity, demonstrates effectiveness, and is capable of identifying faults in rolling bearings.

## Introduction

Rolling bearings are essential to CNC machine tools, significantly affecting CNC’s regular operation. Specifically, the outer ring, inner ring, or rolling part of the rolling bearing is most prone to wear or deformation under high-load operation, affecting the entire production process. Therefore, fault prediction and diagnosis of rolling bearings are significant. Due to the swift advancement of deep learning, data-driven fault diagnosis of rolling bearings has gained increasing popularity. In such strategies, data acquisition is realized by sensors and measured by signal processing methods. Vibration signal analysis is one of the most studied sensing methods at present.

Traditional vibration signal analysis methods rely on manual feature extraction and are difficult to adapt to complex working conditions, such as Fourier transform and wavelet decomposition, Vector Local Characteristic-Scale Decomposition (Vector LCD) [2], fuzzy signal feature fusion technology [3], Principal Component Analysis (PCA), and digital twin and transfer learning [4,5]. Although deep learning models (such as CNN and LSTM) have achieved automatic feature extraction through end-to-end learning, they still face two major challenges in practical industrial applications: High model complexity leads to difficult deployment (for example, the parameter count of ResNet-50 reaches 23M), and it is challenging to systematically analyze the convergence of the model.

The current research on bearing fault diagnosis mainly falls into three categories of methods: Deep learning methods: numerous models have garnered extensive application [6–8], with the main models including CNN, Deep Belief Network (DBN), Recurrent Neural Network (RNN), Gated Recurrent Unit (GRU) [9], Long Short-Term Memory (LSTM) and Resnet [10]. In [11], the authors demonstrated that combining multi-scale CNN and LSTM models can efficiently diagnose bearing faults. CNN can also be combined with a multi-layer perceptron [12] or multi-task model [13]. CNN algorithms mentioned in [14–19] have also been successfully applied to the field of rolling bearings. Concurrently, recent advancements have led to more efficient architectures such as EPyNet, an energy-efficient 1D-CNN architecture, which achieves significant energy reduction and high accuracy on multiple audio emotion recognition datasets while being compatible with CPU and resource-constrained edge devices [20]. In recent years, combining deep learning with attention mechanisms has yielded promising results, with representative methods being Attentive dense CNN [21], Attention-temporal convolutional neural networks (ATCN), Attention-LSTM, Convolutional Bi-Directional LSTM (CBLSTM) [22], 1DCNN-LSTM [23], TCN-BiLSTM, and Attention TCN-BiLSTM [24].These models proposed attained an accuracy surpassing 90% on the CWRU dataset, but it requires GPU acceleration and cannot explain the decision-making basis. [25] combines CNN with the self-attention of Transformer to achieve efficient computing on mobile devices. In the ImageNet classification task, the model with 0.701M parameters was superior to the pure Transformer scheme.

Lightweight models: SVM, KNN, and SCN [26] are computationally efficient, with SCN converging under an inequality supervision mechanism. However, SCN’s sparsity and generalization capabilities require further improvement to facilitate lightweight deployment. Regularization techniques, including *L*_1_, *L*_2_, and smooth *L*_1_ regularization, have been applied to enhance these aspects [27–29]. Among them, $L_{1/2}$ regularization is particularly effective in generating sparser solutions, offering a more accurate model representation while preserving sparsity [30]. The sparsity and generalization performance of $L_{1}/L_{2}$-regularized SCN is insufficient. Further optimization of SCN’s sparsity and generalization is still desirable.

Hybrid architectures combining deep and shallow models: Hybrid models can combine the respective advantages of deep and shallow models in feature extraction and achieving model lightweighting and sparsity. Unfortunately, there are not many cases of fusing deep and shallow models for phased prediction at present. The ones that have been proposed so far include: LSTM-SVM, which uses LSTM for signal prediction followed by SVM for mechanical state diagnosis [31], and the CNN-LSTM-SVM, which extracts signal features via CNN and LSTM before SVM-based fault classification [32]. The average accuracy rate for fault classification achieved by these models exceeds 95.92% on the CWRU dataset.

Current hybrid models are constrained by two main issues: CNN-based approaches are inadequate for representing time-varying fault characteristics like impact periodicity, and the shallow classifiers used lacks the global approximation capabilities like SCN and is not sufficiently sparse.

In response to the above problems, this paper proposes a novel diagnostic framework that integrates LSTM and $L_{1/2}$ regularized SCN. The main contributions include:

1) An $L_{1/2}$ regularization solution algorithm based on the roots of cubic equations is proposed. Theoretically, it is proved that it has a better sparse error bound than *L*_1_ regularization. Construct an incremental supervision mechanism to guarantee that the model converges to a certain extent and simultaneously enhances its feature selection ability. 2) Design a hierarchical feature processing architecture: The LSTM layer extracts temporal features, and the $L_{1/2}$-SCN layer conducts sparse classification. 3) On the CWRU dataset, experimental evaluations demonstrate that the proposed model achieves a 0.64% improvement in average classification accuracy and attains 23.44% model sparsity when compared with state-of-the-art benchmarks including TCN-LSTM, TCN-BiLSTM, ResNet architectures, and other representative methods.

The remainder of this article is organized as follows. The second part introduces the preliminary knowledge about LSTM and SCN. The third part proposes a sparse learning algorithm based on $L_{1/2}$ regularization and then provides the fusion sparse learning algorithm. The fourth part conducts some numerical experiments to verify the effectiveness of the proposed algorithm. Finally, the fifth part summarizes this paper.

## Preliminaries

### Feature extraction method based on LSTM

**Fig 1:**
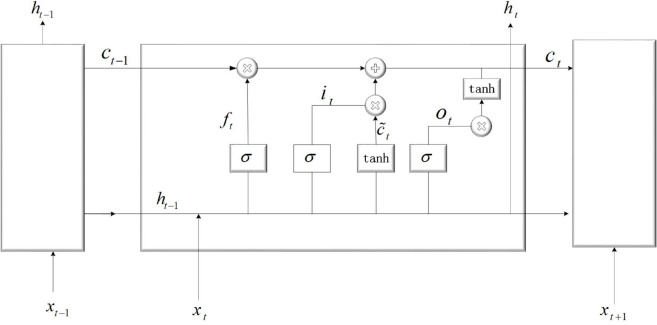
The structure of LSTM.

The state of the system at a certain moment is determined by the combined influence of its past state and the current input. Since the system’s state evolves over time based on these factors, the signals processed by the system are inherently time-dependent. The core design objective of LSTM is to handle sequential data. It can autonomously learn to remember long-term information, forget irrelevant information, and focus on the current input through the forget gate, input gate, and output gate, which makes it well-suited for handling vibration signals with long-term trends and periodic patterns. Meanwhile, LSTM offers a low-attenuation path for gradient backpropagation through cell states and gating mechanisms, thereby effectively alleviating the problem of vanishing gradients. Compared with CNN, which is better at extracting local regional features from signals, LSTM has more advantages in extracting features from sequential data. Fig 1 illustrates the schematic representation of LSTM’s architecture, where *f*_*t*_, *i*_*t*_, and *o*_*t*_ represent the forget gate, input gate, and output gate, respectively. Besides, *c*_*t*_ and *h*_*t*_ represent the state of the cell and hidden layer at time t, σ and tanh are activation functions. LSTM, through its gated architecture, effectively captures both short-term and long-term (*h* and *c*) dependencies in sequential data, making it particularly suitable for tasks like natural language processing and time series forecasting. Specifically, the gating mechanism within LSTM enables data to be added, discarded, and stored within the cell. The forgetting gate *f*_*t*_ processes the forgotten information from *c*_*t*−1_ and preserves the stored data in the current state. The input gate captures the current information, which is then used to compute a candidate cell state *c*_*t*_ combined with the previous cell state *c*_*t*−1_ to generate the new cell state *c*_*t*_. Meanwhile, the output gate *o*_*t*_ determines what part of the cell state *c*_*t*_ is used to create the hidden state *h*_*t*_ for the current time step. The final output represents a comprehensive representation of the current states, and the data flow within LSTM is calculated as follows:

$$f(t)=\sigma (W_{f}\cdot [h_{t-1},x_{t}]+b_{f})$$ (1)

$$i_{t}=\sigma (W_{i}\cdot [h_{t-1},x_{t}]+b_{i})$$ (2)

$$\hat{c_{t}}=tanh(W_{c}\cdot [h_{t-1},x_{t}]+b_{c})$$ (3)

$$c_{t}=f_{t}c_{t-1}+i_{t}c_{t}$$ (4)

$$o_{t}=\sigma (W_{o}\cdot [h_{t-1},x_{t}]+b_{o})$$ (5)

$$h_{t}=o_{t}\cdot tanh(c_{t})$$ (6)

where *W*_*i*_, *W*_*f*_, *W*_*c*_ and *W*_*o*_ represent the input gate, forget gate, current status, and output gate weights, respectively, and *b*_*i*_, *b*_*f*_, *b*_*c*_ and *b*_*o*_ represent the corresponding bias. To improve the learning performance and obtain more specific data features, LSTM is used to extract the time features, and the output h of the hidden layer is used as the data features. The output of LSTM reflects the relevant historical information. Due to its superiority in processing time series data, this paper does not employ a complex deep model for end-to-end fault diagnosis processing. LSTM is selected for the feature extraction stage.

### Principles of stochastic configuration networks

**Fig 2:**
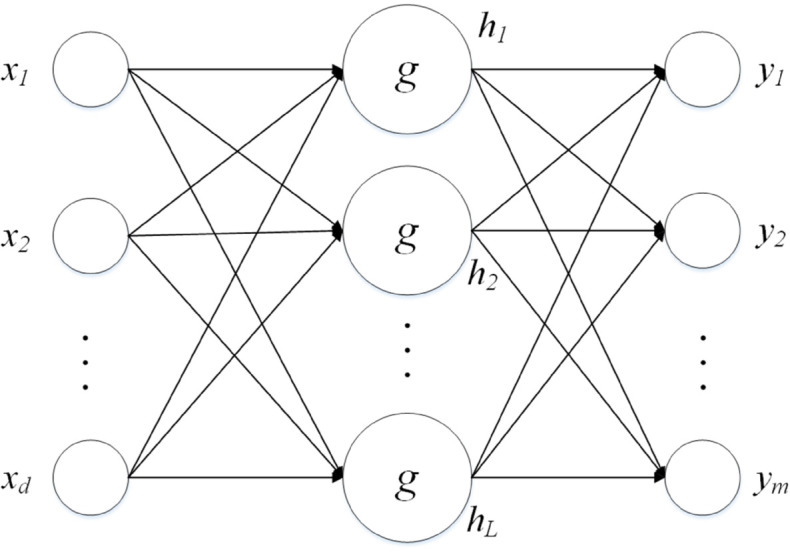
The structure of SCN with L hidden nodes.

Let $X=(x^{1},x^{2},...,x^{N}{)}^{T}$ be the input data, where $x^{i}=(x_{1}^{i},x_{2}^{i},...,x_{d}^{i})$. $T=(t^{1},t^{2},...,t^{N}{)}^{T}$ are the corresponding output data , where $t^{i}=(t_{1}^{i},t_{2}^{i},...,t_{m}^{i})$, *N* signifies the quantity of samples, *d* denotes the dimensionality of input features, and *m* represents the count of output features. The structure of SCN with *L* hidden nodes is depicted in Fig 2.

Let the weights and biases between the input and hidden layer be $W=(w_{1},w_{2},...,w_{L})$, $b=(b_{1},b_{2},...,b_{L})$, where $w_{l}=(w_{l1},w_{l2},...,w_{ld}{)}^{T}$, l=1,2,...,L, $b_{l}\in R$. Then, the output of the L-th hidden node and outputs of all hidden nodes are formulated in (7) and (8).

$$h_{l}=g(Xw_{l}+b_{l})$$ (7)

$$H=(h_{1},h_{2},...,h_{L})=g(XW+b)$$ (8)

where $X\in {\mathbb{R}}^{N\times d}$, $H\in {\mathbb{R}}^{N\times L}$, $W\in {\mathbb{R}}^{d\times L}$, *g* is the activation function. The weight between the hidden and output layer is $\beta =(\beta_{1},\beta_{2},...,\beta_{L}{)}^{T}$, where $\beta \in {\mathbb{R}}^{L\times m}$, $\beta_{l}=(\beta_{l1},\beta_{l2},...,\beta_{lm})$ , the output of SCN is

$$y(L)=(y_{1}(L),y_{2}(L),...,y_{m}(L))=H\beta =\sum_{l=1}^{L}h_{l}\beta_{l}$$ (9)

the error is

$$e_{L}=T-H\beta =(e_{L1},e_{L2},...,e_{Lm})$$ (10)

where $T\in {\mathbb{R}}^{N\times m}$, then the equation $e_{L+1}=e_{L}-h_{L+1}\beta_{L+1}$ can be get based on the equation $y(L+1)=y(L)+h_{L+1}\beta_{L+1}$. The construction process of the model begins with its initialization, setting y(0)=0. Subsequently, *e*_0_ is calculated as T−y(0)=T. When the L-th node is generated, the choice of *w*_*L*_ and *b*_*L*_ follows the inequality supervision mechanism.

$$\sum_{q=1}^{m}\langle e_{L-1,q},h_{L}\rangle^{2}\geq b_{g}^{2}(1-r-\mu_{L})\|e_{L-1}{\|}^{2}$$ (11)

where *e*_*L*−1,*q*_ represents the q-th dimension error after the L-1 hidden node has been configured, *b*_*g*_ is the upper bound of the activation function, *r* is a constant close to 1, and the real number sequence $\left\{\mu_{L}\right\}$ is satisfied $\lim_{L\to +\infty }\mu_{L}=0$. The inequality constraint in Eq (11) forms the theoretical foundation for SCN stability by guaranteeing monotonic error reduction during incremental construction. This inequality supervision is essential because: (i) it ensures each new hidden node decreases the residual error to guarantee the convergence of the network, preventing network overgrowth; (ii) the parameters *r* and μ create a contraction mapping that guarantees convergence. Without this constraint, random node addition could cause oscillating or divergent training behavior. β can be determined by the global least square method by (12).

$$\beta^{*}=\underset{\beta }{argmin}\|H\beta -T{\|}^{2}=H^{†}T$$ (12)

When the first node has been configured (*w*_1_,*b*_1_, and $\beta_{1}$ are determined), the above steps are repeated to gradually increase the nodes and guide the predetermined maximum number or accuracy.

## The fusion sparse learning algorithm

### The sparse learning algorithm of $L_{1/2}$-SCN

The unregularized SCN employs least squares for weight estimation, often resulting in numerical instability and overfitting. While *L*_1_ regularization improves sparsity and reduces model complexity. Theoretical analysis demonstrates that $L_{1/2}$ regularization possesses stronger sparsity-inducing properties than *L*_1_ regularization [30], $L_{1/2}$ regularization strikes an optimal balance between *L*_0_ sparsity and *L*_1_ tractability, and its non-convex formulation better approximates *L*_0_’s sparsity while remaining computationally feasible. Meanwhile, in practical scenarios with limited samples, its adaptive thresholding mechanism provides superior noise-feature discrimination by selectively preserving weak but diagnostically significant fault characteristics.

$L_{1/2}$ regularization is an effective sparsity method that improves the error function of SCN, specifically by adding the $L_{1/2}$ regularization term to the objective function, as presented in (13). Here, λ is the regularization coefficient.

$$\underset{\beta }{min}:\frac{1}{2}\|H\beta -T{\|}^{2}+\lambda \|\beta {\|}_{1/2}^{1/2}$$ (13)

The Admm algorithm is used to solve the $L_{1/2}$ regularization problem, and the specific methods are described below. Construct the optimization problem:

$$min:f(x)+g(\beta )=\frac{1}{2}\|Hx-T{\|}^{2}+\lambda \|\beta {\|}_{1/2}^{1/2}$$ (14)

s.t.x−β=0 (15)

Let $\mu_{1}=\frac{\mu }{\rho }$, the original problem is equivalent to solving the following problem

$$x^{k+1}=\underset{x}{argmin}(f(x)+\frac{\rho }{2}\|x-\beta^{k}+\mu_{1}^{k}{\|}_{2}^{2})$$ (16)

$$\beta^{k+1}=\underset{\beta }{argmin}(g(\beta )+\frac{\rho }{2}\|x^{k+1}-\beta +\mu_{1}^{k}{\|}_{2}^{2})$$ (17)

$$\mu_{1}^{k+1}=\mu_{1}^{k}+x^{k+1}-\beta^{k+1}$$ (18)

solve for (16) and the following equation can be get

$$x^{k+1}=(H^{T}H+\rho I{)}^{-1}(H^{T}T+\rho (\beta^{k}-\mu_{1}^{k}))$$ (19)

Take the derivative of β in formula (17), search the stagnation point, get the equation (20)

$$\rho \beta +\frac{\lambda }{2}\cdot \frac{sign(\beta )}{\sqrt{\left(\beta \right)}}-\rho (x^{k+1}+\mu_{1}^{k})=0$$ (20)

a. If β>0, let $t=\sqrt{\left(|\beta |\right)}$, then formula (20) is converted to (21)

$$t^{3}-(x^{k+1}+\mu_{1}^{k})t+\frac{\lambda }{2\rho }=0$$ (21)

let $m=x^{k+1}\;+\;\mu_{1}^{k}$, $n=\frac{\lambda }{2\rho }$, it can be seen from the cubic equation and the graph form that when the discriminant $\Delta =(\frac{n}{2}{)}^{2}\;-\;(\frac{m}{3}{)}^{3}<0$, namely $(\frac{m}{3}{)}^{3}>(\frac{n}{2}{)}^{2}$, the equation has three unequal real roots. According to Cartan’s formula, the roots of the equation are one negative and two positive, and the largest positive root is the minimum point of (17), as shown in (22) and (23). For the $L_{1/2}$ regularization problem, Xu et al. [30] proved that the objective function is unimodal on the positive real axis, with its unique critical point (the maximum root of the cubic equation) guaranteed to correspond to a local minimum, as verified through second-order convexity analysis.

$$t=2\sqrt{\left(|m|/3\right)}\cdot cos(\frac{\pi }{3}-\frac{\phi }{3})$$ (22)

$$\phi =arccos(\frac{n}{2}\cdot (\frac{|m|}{3}{)}^{\frac{-3}{2}})$$ (23)

therefore

$$\beta =\frac{4|m|}{3}\cdot cos^{2}(\frac{\pi }{3}-\frac{\phi }{3})$$ (24)

b. If β<0, let $t=\sqrt{\left(|\beta |\right)}$, then formula (20) is converted to (25)

$$t^{3}+(x^{k+1}+\mu_{1}^{k})t+\frac{\lambda }{2\rho }=0$$ (25)

In a similar way, when $(\frac{m}{3}{)}^{3}<-(\frac{n}{2}{)}^{2}$

$$\beta =-\frac{4|m|}{3}\cdot cos^{2}(\frac{\pi }{3}-\frac{\phi }{3})$$ (26)

Then the optimal solution of the objective function is

$$\beta^{k+1}=\left\{ \begin{array}{cc} \frac{4|m|}{3}\cdot cos^{2}(\frac{\pi }{3}-\frac{\phi }{3}), & \;\;(\frac{m}{3}{)}^{3}>(\frac{n}{2}{)}^{2}\\ 0, & \;\;others\\ -\frac{4|m|}{3}\cdot cos^{2}(\frac{\pi }{3}-\frac{\phi }{3}), & \;\;(\frac{m}{3}{)}^{3}<-(\frac{n}{2}{)}^{2} \end{array}\right.$$ (27)

The update formula of $\mu_{1}$ can be equivalently converted from (18) to (28).

$$\mu_{1}^{k+1}=\mu_{1}^{k}+x^{k+1}-\beta_{q}^{k+1}$$ (28)

In summary, the sparse learning algorithm of $L_{1/2}$ regularized SCN is given by iterative solution according to formulas (19), (27) and (28).

### Inequality supervision mechanism for $L_{1/2}$-SCN

Analysis the objective function

$$J=\frac{1}{2}\|H\beta -T{\|}^{2}+\lambda \|\beta {\|}_{1/2}^{1/2}$$ (29)

$$=\frac{1}{2}\|e_{L-1}-g_{L}\beta_{L}{\|}^{2}+\lambda \sum (|\beta {|}^{1/2})$$ (30)

$$\frac{\partial J}{\partial \beta_{L}}=-g_{L}(e_{L-1}-g_{L}\beta_{L})+\frac{\lambda }{2}\cdot \frac{sign(\beta_{L})}{\sqrt{(}|\beta_{L}|)}=0$$ (31)

in the same way as the solution for (20), let $ieql=(\frac{\langle e_{L-1},g_{L}\rangle }{3g_{L}^{2}}{)}^{3}$,$ieqr=2(\frac{\lambda }{4g_{L}^{2}}{)}^{2}$,we can get

$$\beta_{L}=\left\{ \begin{array}{cc} \frac{4}{3}|\frac{\langle e_{L-1},g_{L}\rangle }{g_{L}^{2}}|\cdot cos^{2}(\frac{\pi }{3}-\frac{\phi }{3}), & ieql>ieqr\\ 0, & |ieql|\leq ieqr\\ -\frac{4}{3}|\frac{\langle e_{L-1},g_{L}\rangle }{g_{L}^{2}}|\cdot cos^{2}(\frac{\pi }{3}-\frac{\phi }{3}), & ieql<-ieqr \end{array}\right.$$ (32)

In SCN, the choice of w and b need to satisfy the inequality $\|e_{L-1}{\|}^{2}-\|e_{L}{\|}^{2}\geq (1-r-\mu )\|e_{L-1}{\|}^{2}$, namely

$$2\langle e_{L-1},g_{L}\rangle \beta_{L}-\beta_{L}^{2}g_{L}^{2}\geq (1-r-\mu )\|e_{L-1}{\|}^{2}$$ (33)

Let

$$v_{1}=\frac{|\langle e_{L-1},g_{L}\rangle |}{g_{L}^{2}}\cdot cos^{2}(\frac{\pi }{3}-\frac{\phi }{3})$$ (34)

$$v_{2}=\frac{8}{3}\langle e_{L-1},g_{L}\rangle$$ (35)

$$v_{3}=\frac{16}{9}|\langle e_{L-1},g_{L}\rangle |\cdot cos^{2}(\frac{\pi }{3}-\frac{\phi }{3})$$ (36)

An inequality supervision mechanism for $L_{1/2}$-SCN is obtained by substituting the expression for $\beta_{L}$ into (33), as shown in (37) and (38). The following conclusions can be drawn:

if

$$\left(\frac{\langle e_{L-1},g_{L}\rangle }{3g_{L}^{2}}{)}^{3}>2(\frac{\lambda }{4g_{L}^{2}}{)}^{2}\right)$$ (37)

the inequality supervision mechanism is:

$$v_{1}(v_{2}-v_{3})\geq (1-r-\mu_{L})\|e_{L-1}{\|}^{2},$$ (38)

and if

$$\left(\frac{\langle e_{L-1},g_{L}\rangle }{3g_{L}^{2}}{)}^{3}<-2(\frac{\lambda }{4g_{L}^{2}}{)}^{2}\right)$$ (39)

the inequality supervision mechanism is:

$$-v_{1}(v_{2}+v_{3})\geq (1-r-\mu_{L})\|e_{L-1}{\|}^{2},$$ (40)

Therefore, new hidden nodes are incrementally added when either condition (37) or (39) is satisfied, strictly following the inequality constraints specified in (38) or (40) respectively. If neither condition is met, according to (32), the corresponding weight is set to zero. It is noteworthy that the inequality supervision mechanism proposed above enables the model to converge to a certain degree. Nevertheless, during the sparse - processing procedure, β is set to zero without fulfilling the inequality constraints. Formula (32), (37)–(40) reveal the contradictions inherent in these two aspects. Consequently, in practical applications, it is imperative to strike a balance between sparsity and model accuracy. The *L*_1/2_-SCN proposed in this section theoretically analyzes its own convergence and updates the original inequality supervision mechanism. This update allows the algorithm to offer a sparser model representation, which is advantageous for actual fault identification and classification tasks. In the entire fault diagnosis process, the algorithm can take over the feature extraction task from the previous stage to facilitate fault type identification. The algorithm flow of *L*_1/2_-SCN is shown in Algorithm 1.

### Fusion sparse learning algorithm

**Fig 3:**
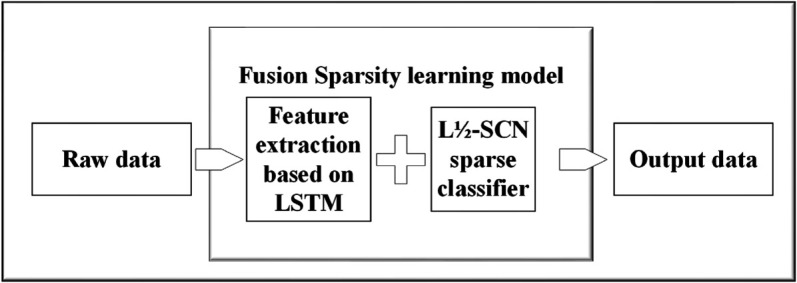
The fault identification method utilizing fusion sparse learning model.

Massive information, inherent noise, temporal dependencies, and pronounced periodicity typically govern signal data. Utilizing a singular model for learning may hinder the thorough examination of the underlying patterns within the data. To confront the intricacies arising from voluminous datasets and vague features, this study employs LSTM to extract temporal features. Subsequently, these features are input for sparse learning via the $L_{1/2}$-SCN model, enhancing its performance and resulting in a sparse structural representation. Fig 3 depicts the architecture of the fusion model, while the detailed algorithmic steps are outlined in Algorithm 2.

**Algorithm 1 Incremental node addition with adaptive regularization.**

**Require:**

$𝐗\in {\mathbb{R}}^{N\times d}$ ▷ Input data matrix (N samples × d features)

$𝐓\in {\mathbb{R}}^{N\times m}$ ▷ Target matrix

$\xi =10^{-2}$ ▷ Residual error tolerance (convergence threshold)

$L_{\text{max}}=500$ ▷ Maximum hidden nodes

$T_{\text{max}}=30$ ▷ Max attempts per regularization parameter

**Ensure:**

$\beta =[\beta_{1},\beta_{2},...,\beta_{L}]$, ▷ Output weights

$w^{*}=[w_{1}^{*},w_{2}^{*},...,w_{L}^{*}]$, $b^{*}=[b_{1}^{*},b_{2}^{*},...,b_{L}^{*}]$ ▷ Optimal node parameters

1: Initialize: $e_{0}\leftarrow 𝐓$,r←0.9 ▷ Initialization

2: Set regularization grid: γ←{1,5,10,20,30} ▷ Λ search

range

3: **while** $L\leq L_{\text{max}}$ **and** $\|e_{L}{\|}_{2}>\xi$ **do**

4:   **for** Λ∈γ **do** ▷ Adaptive node generation

5:    **for** *k* = 1 to $T_{\text{max}}$

6:     Sample $w_{L}\sim 𝒰(-\Lambda ,\Lambda )$, $b_{L}\sim 𝒰(-\Lambda ,\Lambda )$ ▷ Random

projection

7:     **if** Inequality (32) satisfied **then**

8:      $W\leftarrow W\cup \{w_{L}\}$, ▷ Archive valid nodes

9:     **end if**

10:    **end for**

11:    **if** W≠∅ **then**

12:     Select $\left(w_{L}^{*},b_{L}^{*}\right)$ maximizing formula (33) ▷ Node

selection

13:     **break** (textbfgoto (15))

14:    **else**

15:     Adjust r←r+τ, τ~𝒰(0,1−r),Return 4 ▷ Relax

supervision

16:    **end if**

17:   **end for**

18:   Compute $\beta^{*}$ via Eq (19), (27), (28) ▷ Least squares

solution

19:   Update residual: $e_{L}\leftarrow e_{L-1}-\beta_{L}h_{L}^{*}$, $h_{L}^{*}=[g_{1}^{*},g_{2}^{*},...,g_{L}^{*}]$

▷ Error correction

20:   L←L+1

21: **end while**

**Algorithm 2**

1: Set input-output data pair (*X*,*Y*);

2: Initialize the parameters of LSTM, including learning rate, optimizer, activation function, weights and biases $W_{i},W_{f},W_{c},W_{o},b_{i},b_{f},b_{c},b_{o}$; Optimization: SGDM with learning rate η=0.2, momentum γ=0.5

3: Calculate the outputs of LSTM according to Formula (1)-(6), and constantly update the weights by BP algorithm to get the output *h*;

4: Normalize *h* to $h^{\prime }$;

5: Input $h^{\prime }$ into $L_{1/2}$-SCN classifier and perform calculation according to Algorithm 1; ▷ Sigmoid activation function for all hidden nodes

6: Return the outputs of $L_{1/2}$-SCN: $y(L)=(y_{1}(L),y_{2}(L),...,y_{m}(L))$.

Standardized Feature Fusion Pipeline is as follows:

(1) Temporal Feature Extraction The original input data $X\in {\mathbb{R}}^{N\times d}$ undergoes feature extraction through a single-layer LSTM network configured with hidden units:

$$H\_LSTM_{t}=LSTM(X;W_{h},U_{h},b_{h}),\;\;\;\;H\_LSTM_{t}\in {\mathbb{R}}^{N\times h}$$ (41)

where *W*_*h*_, *U*_*h*_ and *b*_*h*_ denote the input weights, recurrent weights, and bias terms, respectively.

(2) Standardize the output $H\_LSTM_{t}$ of the LSTM layer. (The numerical range of LSTM hidden states is influenced by both the input data’s physical dimensions and the activation function, potentially resulting in magnitude variations across different samples. Therefore, the data needs to be normalized)

(3) The standardized data is taken as input and entered into the $L_{1/2}$-SCN classifier for learning and training to obtain classification.

The algorithm proposed above constitutes a two-stage hybrid approach. As an end-to-end learning framework, during the classification phase, the parameter selection for the $L_{1/2}$-SCN model is guided by a rigorous inequality supervision mechanism, and its convergence properties have been analyzed. Consequently, in comparison to other deep learning models, the proposed algorithm exhibits mathematical interpretability with respect to its convergence behavior. This is also the reason for this paper emphasizing the proposed model’s some interpretability. However, we admit that the selection of model parameters is still random, and it is not a deterministic mathematical model that can be analyzed in terms of its underlying mechanism.

## Numerical experiments

This section employs $L_{1/2}$-SCN on benchmark datasets to demonstrate its effectiveness in sparsity and generalization. The fusion algorithm is then used to determine the fault type based on the Case Western Reserve University dataset. Meanwhile, we also designed a comparative experiment using $L_{1/2}$-SCN without a feature extraction process to illustrate the effectiveness of feature extraction.

### Experiments based on the benchmark datasets

The subsequent experiments rely on the Iris, Wine, Mnist, Prostate, and Dee datasets from UCI Machine Learning Repository. The first three datasets are used for classification, while the remaining datasets are used for regression. Table 1 summarizes the attributes of these datasets. In [27] and [28], the authors introduced SCN with *L*_2_ and *L*_1_ regularization terms, respectively, denoted as RSCN (Regularized SCN) and PSCN (Parsimonious SCN). The generalization performance and sparsity of $L_{1/2}$-SCN will be compared with RSCN, PSCN, and SCN. However, RSCN and SCN do not possess sparse capabilities, so $L_{1/2}$-SCN will primarily be compared with PSCN regarding sparsity. Table 2 reports the parameters of all models, where *C* represents the regularization parameter of RSCN.

**Table 1:** Attributes of dataset.

Dataset	Samples size	Training samples	Test samples	Input Features	Output Features	Attribute
Iris	120	90	30	4	3	Classification
Wine	178	148	30	13	3	Classification
Mnist	70000	60000	10000	784	10	Classification
Prostate	97	67	30	8	1	Regression
Dee	365	300	65	6	1	Regression

**Table 2:** Parameters setting.

Dataset	Hidden nodes	λ	*C*	Iterations of ADMM
Iris	40	0.005	2^5^	1000
Wine	20	0.005	2^10^	1000
Mnist	200	0.0005	2^5^	100
Prostate	200	0.0005	2^10^	1000
Dee	70	0.005	2^10^	2000

Let *N*_*R*_ represent the samples that is correctly classified, *N*_*T*_ represent the total number of samples, the classification accuracy is defined as follows.

$$ACC=\frac{N_{R}}{N_{T}}$$ (42)

Define the root mean square error(RMSE) as follows.

$$RMSE=\sqrt{\left(\frac{1}{N_{T}}\sum_{i=1}^{N_{T}}(t^{i}-y^{i}{)}^{2}\right)}$$ (43)

where *t^i^* is the target output of the i-th sample, while *y^i^* is the network output. Let $\beta_{total}$ represent the number of weights between the hidden layer and output layer ($L_{1/2}$-SCN, PSCN, RSCN and SCN), and let *D* represent the number of zero weights among them, and define sparsity *Z* as follows:

$$Z=\frac{\beta_{zero}}{\beta_{total}}$$ (44)

The regularization coefficient λ was examined via grid search. When λ was set to the values presented in Table 2, the optimal sparsity-precision trade-off was achieved. The number of hidden nodes was incrementally increased from one to the values listed in Table 2. Beyond these node counts, model performance remained stable.

Table 3 evaluates the models on the first three datasets based on classification accuracy and the last two datasets using the RMSE criterion. Therefore, the data in the table is described by ’Accuracy or RMSE’.

**Table 3:** Performance comparison on the test set.

Dataset	Accuracy or RMSE			
	$L_{1/2}$-SCN	PSCN	RSCN	SCN
Iris	**96.33%**	93.00%	93.50%	90.50%
Wine	**99.67%**	99.50%	97.75%	96.50%
Mnist	**90.10%**	89.99%	89.58%	89.68%
Prostate	**0.2269**	0.2409	0.2373	0.9471
Dee	0.0932	0.0892	0.0892	0.0890

**Fig 4:**
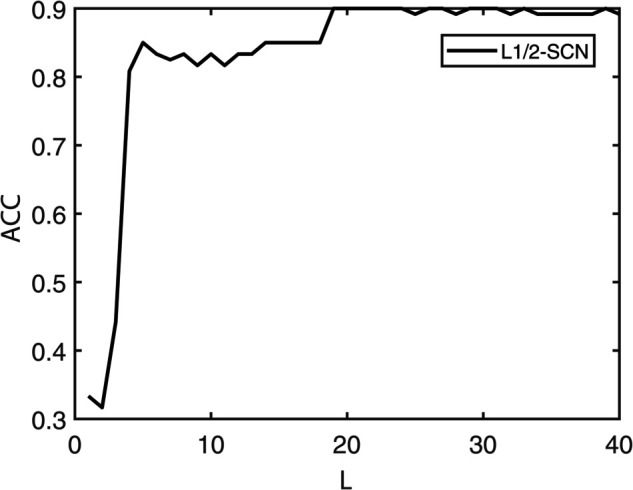
Convergence of L1/2-SCN: Training ACC achieve 98% with 40 nodes (Iris).

**Fig 5:**
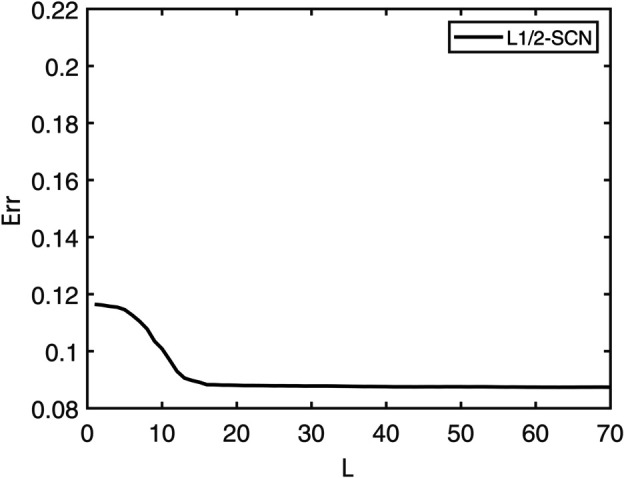
Convergence of L1/2-SCN: training loss drops below 0.09 with 70 nodes (Prostate).

**Fig 6:**
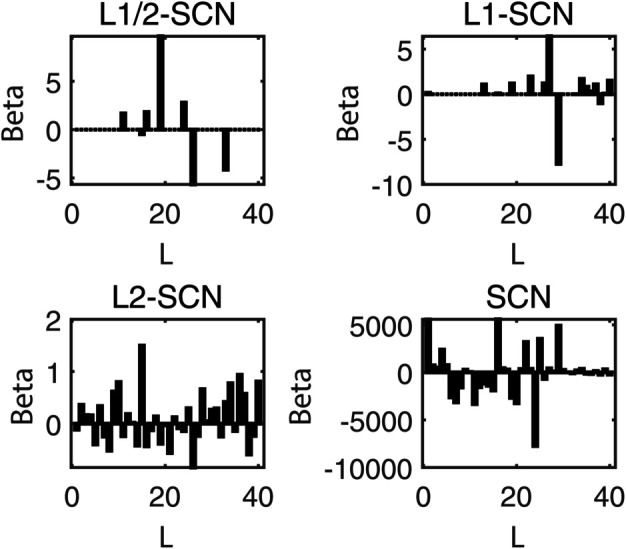
Sparsity pattern contrast: L1/2-SCN achieves 76.66% zero weights (Iris).

**Fig 7:**
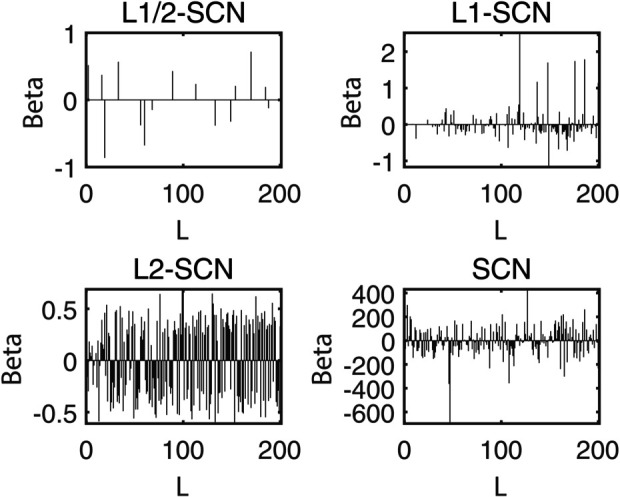
Sparsity pattern contrast: L1/2-SCN achieves 92.50% zero weights (prostate).

Table 3 reports the results of each test set, and Table 4 presents the sparsity of each model. Table 3 highlights that $L_{1/2}$-SCN exhibits superior generalization performance on most datasets, and its sparsity remains superior even when the generalization performance is comparable. In both the Iris and Prostate benchmark experiments, the classification accuracy progressively improves with increasing numbers of hidden nodes, while the regression error exhibits a consistent decline. This phenomenon demonstrates the critical role of node quantity in model capacity (Figs 4 and 5). Figs 6 and 7 demonstrate the weights distribution of the four models. Table 4 presents the sparsity, indicating that the sparsity degree of $L_{1/2}$-SCN is higher than that of PSCN, verifying that $L_{1/2}$ regularization leads to better sparsity.

**Table 4:** Sparsity of each model (Ratio of zero weights between the hidden layer and the output layer).

	$L_{1/2}$-SCN	PSCN	RSCN	SCN
Iris	**76.66%**	46.67%	0	0
Wine	**21.66%**	8.33%	0	0
Mnist	**4.70%**	0.05%	0	0
Prostate	**92.5%**	36.50%	0	0
Dee	**91.42%**	71.43%	0	0

Notably, on the Mnist dataset, the model achieves increased accuracy as the number of hidden nodes rises to 200. Both $L_{1/2}$-SCN and PSCN demonstrate excellent performance on the Wine dataset, with an accuracy of less than 100% only one or two times out of 20 experiments. Notably, $L_{1/2}$-SCN excels in sparsity despite the similar classification capabilities of the two models.

### Fault diagnosis experiment of rolling bearings

#### Experimental methodology.

**Fig 8:**
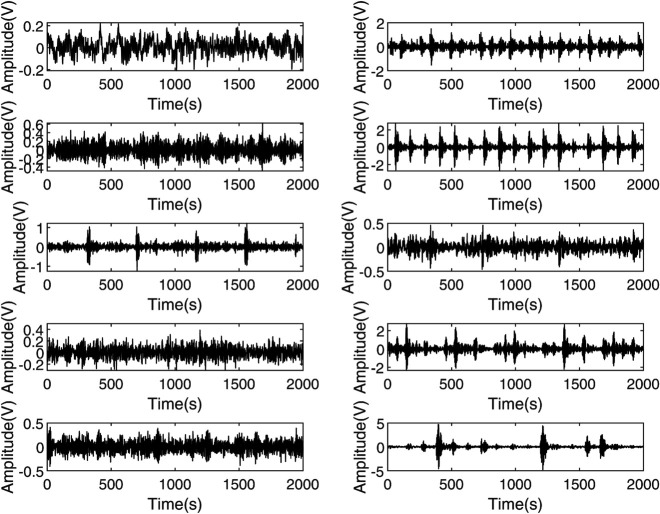
Fault data distribution diagram of the drive end of the motor housing.

In this section, the performance of the proposed model is verified using the rolling bearing failure dataset of Western Reserve University in the United States. The rolling bearing fault experiment introduces varying-sized fault points into the three parts of the bearing. Precisely, accelerometers are placed on the bearing, the motor’s driving terminus, and the fan-facing extremity to collect vibration data. Data from the motor housing drive end are also recorded at a sampling rate of 12,000 samples per second. Under a 12 kHz sampling rate, 12,000 samples correspond to a 1-second time duration, which fully encompasses the characteristic periodicity of typical bearing fault frequencies. This paper selects standard data and nine types of fault data spanning four cases of motors ranging from 0 to 3 horsepower (Cases 1 through 4). The fault points on the outer ring are located at 6 o’clock. The specific fault classifications are detailed in Table 5, and Fig 8 illustrates the vibration signals for nine different faults and normal operating states.

**Table 5:** Classification of faults in motor housing driver-end data (case 4).

Status	Description
1	Manufacture a fault of 0.007 inches on the ball
2	Manufacture a fault of 0.014 inches on the ball
3	Manufacture a fault of 0.021 inches on the ball
4	Manufacture a fault of 0.007 inches in the inner ring
5	Manufacture a fault of 0.014 inches in the inner ring
6	Manufacture a fault of 0.021 inches in the inner ring
7	Manufacture a fault of 0.007 inches of the bearing outer ring at 6 o’clock
8	Manufacture a fault of 0.014 inches of the bearing outer ring at 6 o’clock
9	Manufacture a fault of 0.021 inches of the bearing outer ring at 6 o’clock
10	Normal state

**Fig 9:**
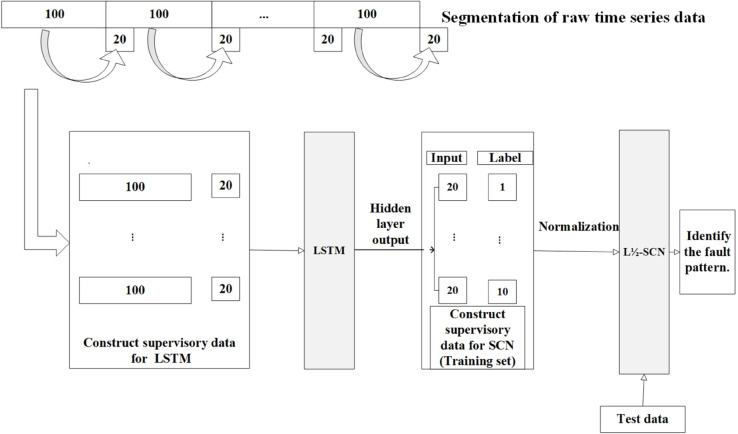
The processing flow of rolling bearing fault dataset.

The specific experimental process is as follows. Step 1. Data Processing: The raw data is initially aligned to ensure consistency in length. For each category, the first 120,000 data points are selected. The signal data of length 120,000 is then divided into a matrix of 1200x100(The segment length of 100 points was determined through time-frequency analysis of bearing vibration characteristics), interpreted as 1200 samples. The supervised learning data for LSTM is constructed by considering the following 20 data points as their corresponding outputs(Through random forest MDI evaluation, the top 20 features are identified as critical discriminators, collectively accounting for 93.5% (95% CI: ±2.1%) of the importance weight), every 100 data points. Step 2. Feature extraction: LSTM extracts 1200*20 features for each category. Step 3. Dataset Splitting: The 1200 samples from each category in Step 2 are utilized for the second-stage experiment. One thousand samples are randomly selected to form the training set, while the remaining 200 constitute the test set. Consequently, the total number in the training and test sets for the second-stage experiment is 10,000 and 2,000, respectively. Step 4. The feature data obtained in Step 3 is normalized and then input into the $L_{1/2}$-SCN for classification, where the output represents the fault category. Fig 9 outlines the processing flow.

The fault identification ability of the proposed method is compared against Attention-TCN, Attention-BiLSTM, TCN-BiLSTM, Attention-TCN-BiLSTM, GRU, Resnet and TCN-Transformer models.

#### Evaluation indexes and results.

The evaluation indicators are Test ACC, Precision, Recall, F1, AUC, ROC curve, PR curve. Considering binary classification, for example, TP signifies the number of True Positives, FP denotes the quantity of False Positives, and FN represents the number of False Negatives. These metrics are defined based on the TP, FP, TN, and FN.

$$Precision=\frac{TP}{TP+FP}$$ (45)

$$Recall=\frac{TP}{TP+FN}$$ (46)

$$F1=\frac{2Precision*Recall}{Precision+Recall}$$ (47)

$$AUC=\frac{TP+TN}{TP+TN+FP+FN}$$ (48)

To ensure a comprehensive evaluation, compare LSTM-L1/2-SCN against a diverse set of benchmarks, which are selected to represent different architectural paradigms in time-series modeling and fault diagnosis.

(1)Hybrid Models: Attention-TCN, Attention-BiLSTM, TCN-BiLSTM, and Attention-TCN-BiLSTM , which capture complex temporal dynamics by the trend of combining convolutional, recurrent and attention models.

(2)Sequential Model: GRU is selected as a simple basic baseline as recurrent neural networks out of these neural network types are known to efficiently work with sequential data.

(3)Deep Residual Architecture: ResNet, a model constructed for computer vision, is used to benchmark against a generic architecture that can learn complex hierarchical representations.

(4)Advanced Transformer-based Architecture: The TCN-Transformer model is selected for a contrastive impact with the long-range properties of a TCN and the global context learning capability of the Transformer, and is one of advanced architectures.

This selection guarantees that the proposed model is evaluated across a wide spectrum of technical routes, thereby providing a holistic demonstration of its performance.

Following the LSTM feature extraction, Tables 6 to 9 compare the performance between LSTM-$L_{1/2}$-SCN and Attention-TCN, Attention-BiLSTM, TCN-BiLSTM, Attention-TCN-BiLSTM, GRU, Resnet and TCN-Transformer. In the experiments, the procedure was executed 50 times.

**Table 6:** Comparison of experimental results (Case 1).

Model	Evaluation indexes (%, Mean ± 95% CI)				
	Test ACC	Precision	Recall	Macro F1	Macro AUC
LSTM-$L_{1/2}$-SCN	**0.9728±0.0096**	**0.9729±0.0096**	**0.9728±0.0096**	**0.9725±0.0100**	**0.9989±0.0003**
TCN-BiLSTM	0.9705±0.0008	0.9708±0.0008	0.9705±0.0008	0.9704±0.0008	0.9265±0.0022
Attention-TCN-BILSTM	0.9706±0.0009	0.9708±0.0008	0.9706±0.0009	0.9708±0.0008	0.9348±0.0017
Attention-TCN	0.9546±0.0012	0.9547±0.0012	0.9546±0.0012	0.9544±0.0013	0.9558±0.0013
Attention-BILSTM	0.9341±0.0012	0.9343±0.0012	0.9341±0.0012	0.9336±0.0012	0.9100±0.0008
GRU	0.8673±0.0014	0.8663±0.0014	0.8673±0.0014	0.8641±0.0016	0.9852±0.0002
Resnet	0.9509±0.0127	0.9552±0.0127	0.9509±0.0127	0.9507±0.0131	0.9964±0.0032
TCN-Transformer	0.9642±0.0017	0.9649±0.0014	0.9642±0.0017	0.9640±0.0017	0.8805±0.0047

**Table 7:** Comparison of experimental results (Case 2).

Model	Evaluation indexes (%, Mean ± 95% CI)				
	Test ACC	Precision	Recall	Macro F1	Macro AUC
LSTM-$L_{1/2}$-SCN	**0.9751±0.0086**	**0.9750±0.0087**	**0.9751±0.0086**	**0.9748±0.0089**	**0.9991±0.0003**
TCN-BILSTM	0.9706±0.0009	0.9710±0.0008	0.9706±0.0008	0.9706±0.0009	0.9185±0.0026
Attention-TCN-BILSTM	0.9706±0.0008	0.9710±0.0009	0.9706±0.0008	0.9705±0.0009	0.9197±0.0022
Attention-TCN	0.9537±0.0013	0.9538±0.0013	0.9537±0.0013	0.9534±0.0013	0.9554±0.0013
Attention-BILSTM	0.9383±0.0010	0.9385±0.0011	0.9383±0.0010	0.9379±0.0010	0.9075±0.0010
GRU	0.8687±0.0013	0.8671±0.0013	0.8687±0.0013	0.8655±0.0014	0.9852±0.0002
Resnet	0.9559±0.0109	0.9572±0.0105	0.9559±0.0109	0.9557±0.0108	0.9976±0.0010
TCN-Transformer	0.9662±0.0014	0.9667±0.0014	0.9662±0.0014	0.9660±0.0015	0.8826±0.0043

**Table 8:** Comparison of experimental results (Case 3).

Model	Evaluation indexes (%, Mean ± 95% CI)				
	Test ACC	Precision	Recall	Macro F1	Macro AUC
LSTM-$L_{1/2}$-SCN	**0.9710±0.0076**	**0.9712±0.0077**	**0.9710±0.0076**	**0.9708±0.0090**	**0.9978±0.0004**
TCN-BILSTM	0.9617±0.0011	0.9629±0.0008	0.9617±0.0011	0.9617±0.0010	0.9500±0.0017
Attention-TCN-BILSTM	0.9638±0.0007	0.9643±0.0007	0.9638±0.0007	0.9637±0.0007	0.9489±0.0013
Attention-TCN	0.9496±0.0010	0.9500±0.0009	0.9496±0.0010	0.9495±0.0009	0.9684±0.0011
Attention-BILSTM	0.9459±0.0010	0.9463±0.0009	0.9459±0.0010	0.9457±0.0010	0.908±0.0017
GRU	0.9248±0.0013	0.9256±0.0012	0.9248±0.0013	0.9244±0.0014	0.9939±0.0001
Resnet	0.9553±0.0210	0.9565±0.0182	0.9553±0.0210	0.9547±0.0222	0.9976±0.0014
TCN-Transformer	0.9616±0.0023	0.9627±0.0021	0.9616±0.0023	0.9615±0.0076	0.9051±0.0041

**Table 9:** Comparison of experimental results (Case 4).

Model	Evaluation indexes (%, Mean ± 95% CI)				
	Test ACC	Precision	Recall	Macro F1	Macro AUC
LSTM-$L_{1/2}$-SCN	**0.9680±0.0091**	**0.9685±0.0092**	**0.9680±0.0091**	**0.9679±0.0100**	0.9899±0.0003
TCN-BILSTM	0.9624±0.0009	0.9628±0.0007	0.9625±0.0009	0.9624±0.0009	0.9195±0.0019
Attention-TCN-BILSTM	0.9626±0.0008	0.9630±0.0008	0.9626±0.0008	0.9625±0.0008	0.9295±0.0017
Attention-TCN	0.9517±0.0009	0.9519±0.0009	0.9517±0.0009	0.9516±0.0009	0.9553±0.0016
Attention-BILSTM	0.9285±0.0012	0.9287±0.0010	0.9285±0.0012	0.9280±0.0011	0.9004±0.0016
GRU	0.8615±0.0015	0.8636±0.0016	0.8615±0.0015	0.8599±0.0016	0.9842±0.0002
Resnet	0.9375±0.0323	0.9414±0.0280	0.9375±0.0323	0.9377±0.0317	**0.9966±0.0020**
TCN-Transformer	0.9609±0.0013	0.9616±0.0012	0.9609±0.0013	0.9607±0.0013	0.8806±0.0048

The above results show that Attention - TCN - BiLSTM is a suboptimal model. To verify the significance of the proposed model in terms of performance comparison, a paired t-test was conducted between the proposed model and Attention - TCN - BiLSTM. Table 10 presents paired t-test results of $L_{1/2}$-SCN and Attention-TCN-BiLSTM.

**Table 10:** Paired t-test results of $L_{1/2}$-SCN vs. Attention TCN-BiLstm (Case 1).

Statistical metric	t-value	p-value
Test ACC	3.8833	0.0003
Precision	3.4725	0.0011
Recall	3.8833	0.0003
Macro F1	3.9434	0.0003
Macro AUC	79.0984	0.0000

**Fig 10:**
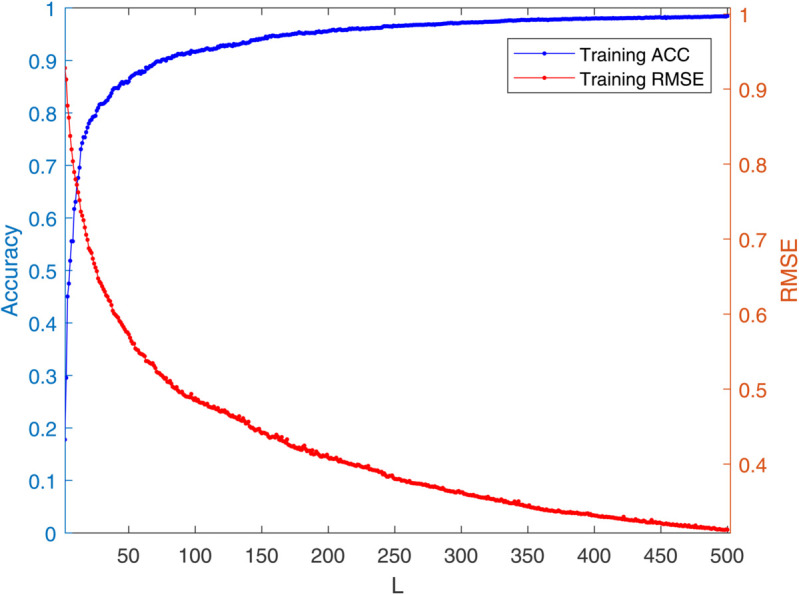
Convergence of L1/2-SCN: training accuracy exceeds 98% with 500 hidden nodes in Case 1.

**Fig 11:**
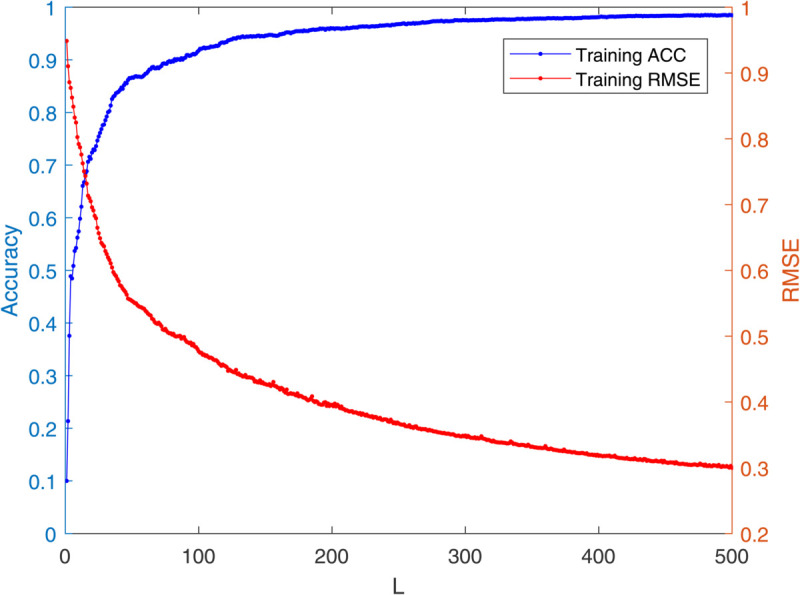
Training accuracy exceeds 98% with 500 hidden nodes in Case 2.

**Fig 12:**
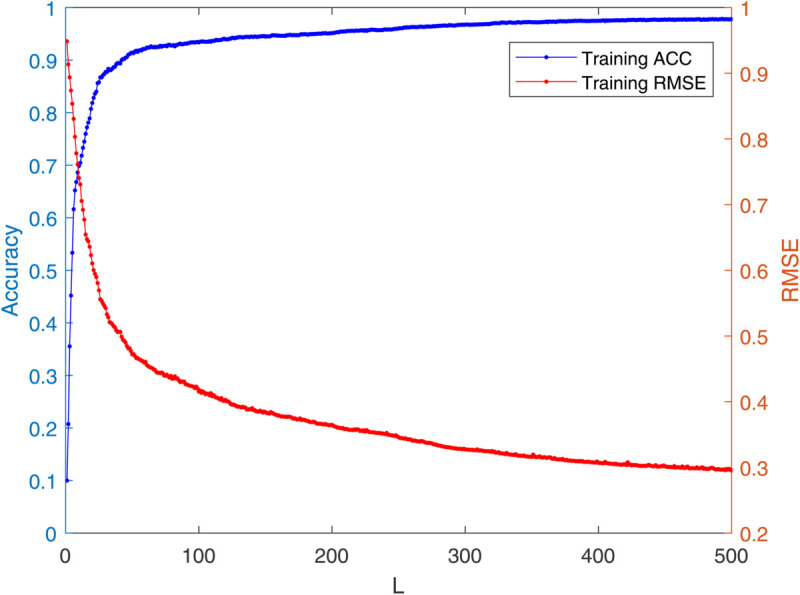
Training accuracy exceeds 98% with 500 hidden nodes in Case 3.

**Fig 13:**
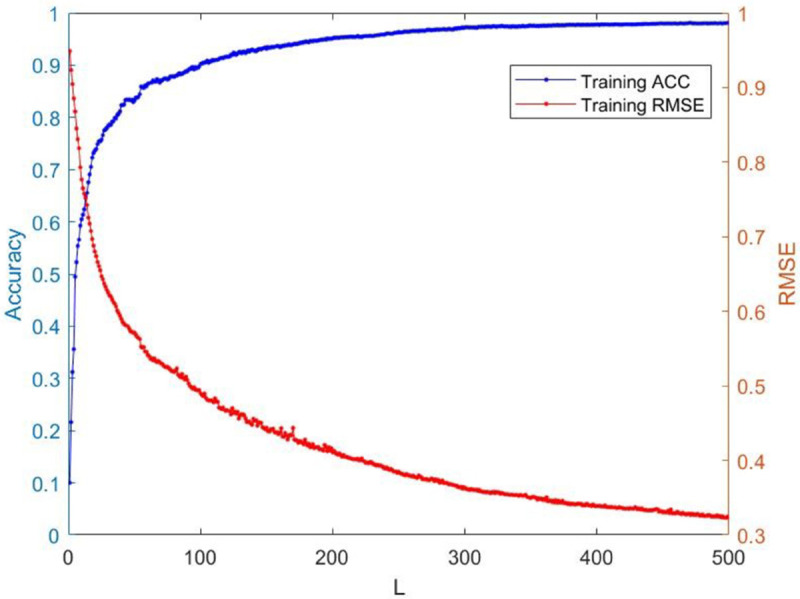
Training accuracy exceeds 98% with 500 hidden nodes in Case 4.

**Fig 14:**
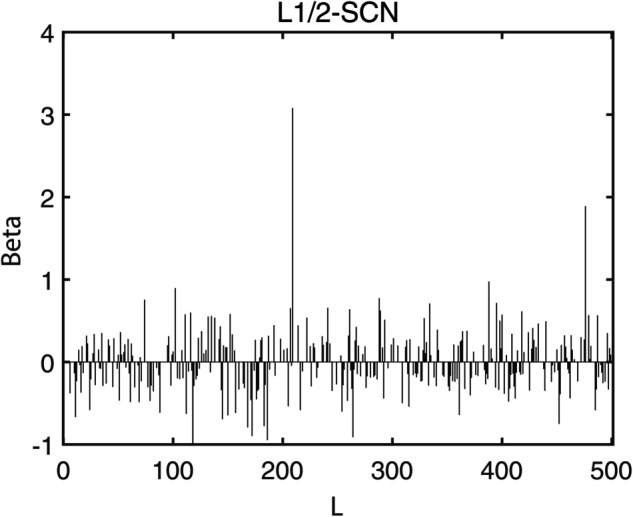
Sparsity pattern contrast: L1/2-SCN achieves 24.24% zero weights (Case 1).

**Fig 15:**
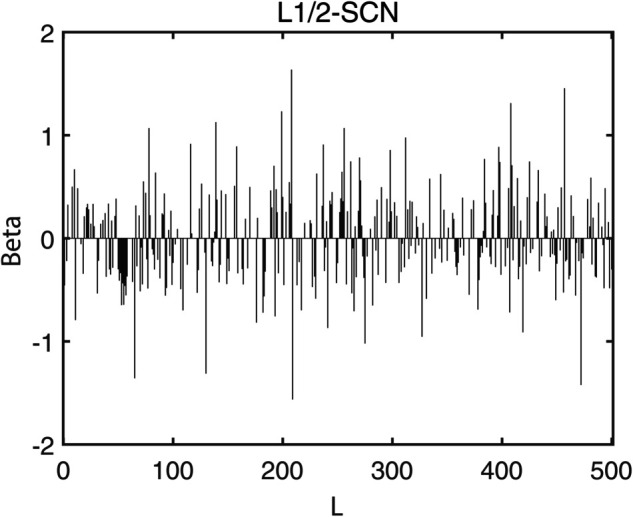
Sparsity pattern contrast: L1/2-SCN achieves 23.88% zero weights (Case 2).

**Fig 16:**
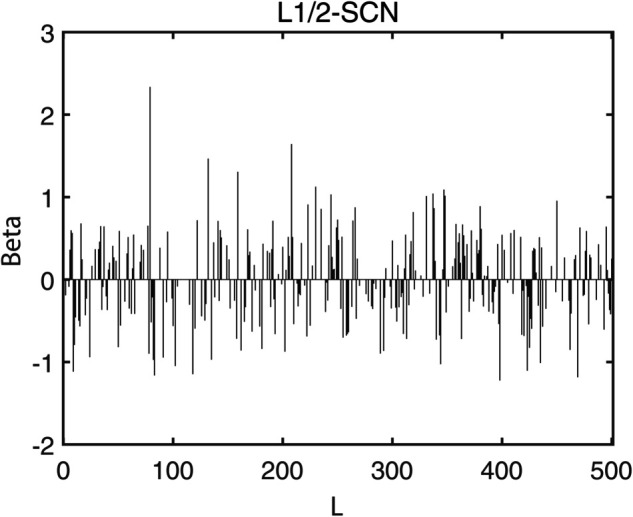
Sparsity pattern contrast: L1/2-SCN achieves 29.39% zero weights (Case 3).

**Fig 17:**
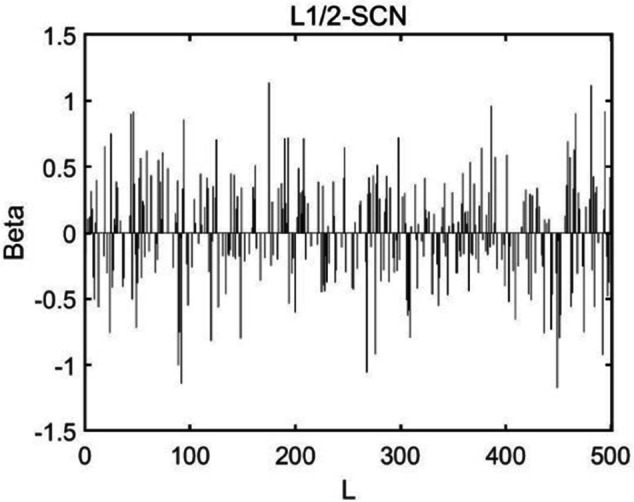
Sparsity pattern contrast: L1/2-SCN achieves 24.72% zero weights (Case 4).

**Fig 18:**
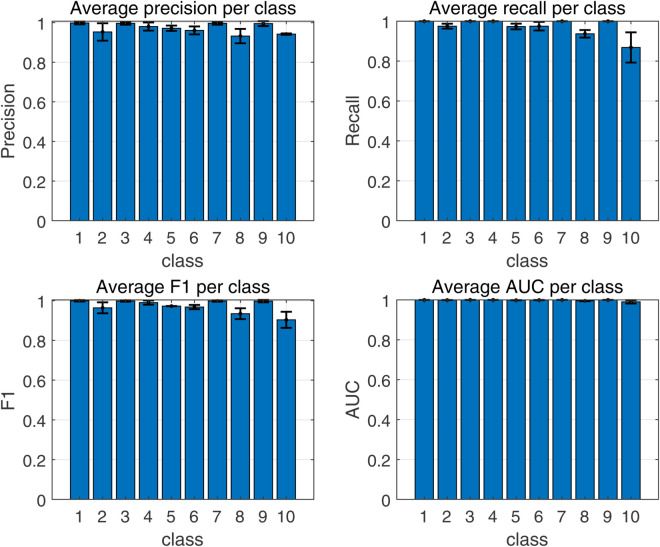
Average metric per class (Case 1).

**Fig 19:**
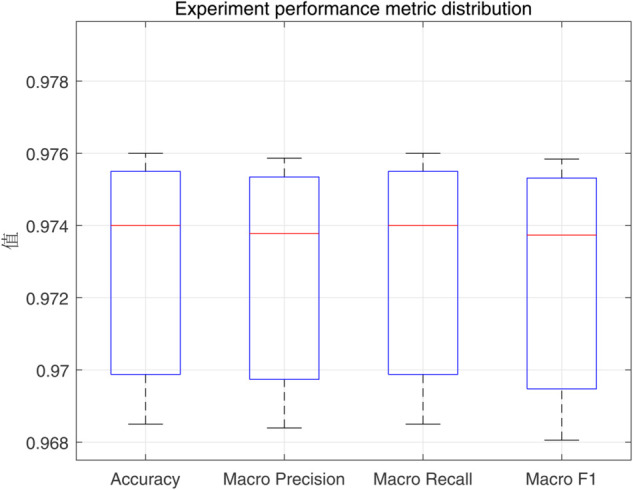
Experiment performance metric distribution (Case 1).

**Fig 20:**
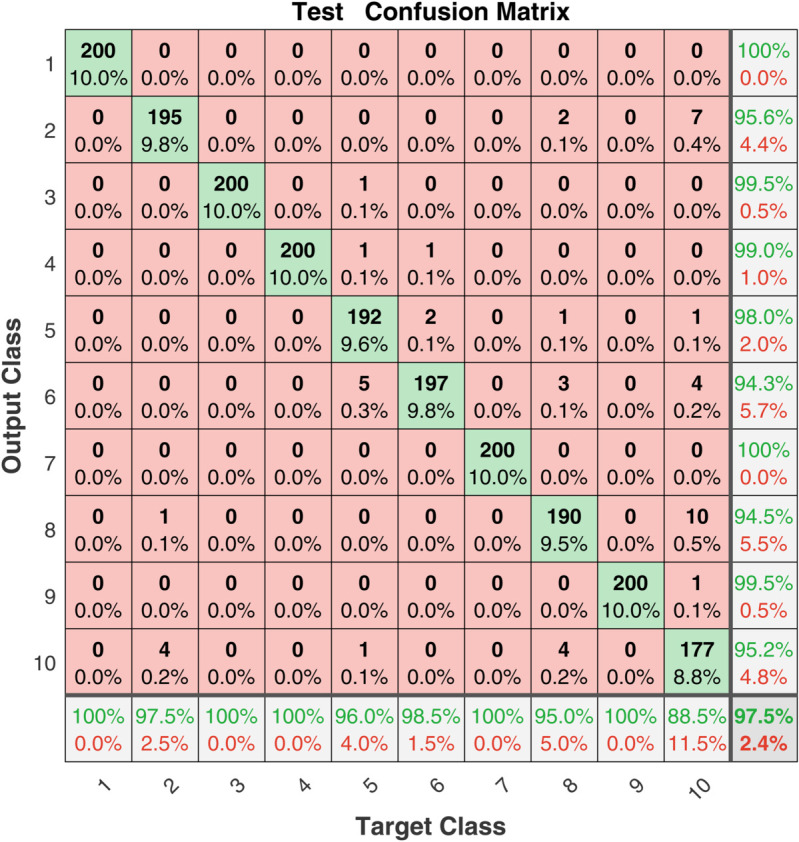
The confusion matrix on the test set for a specific experiment (Case 1).

**Fig 21:**
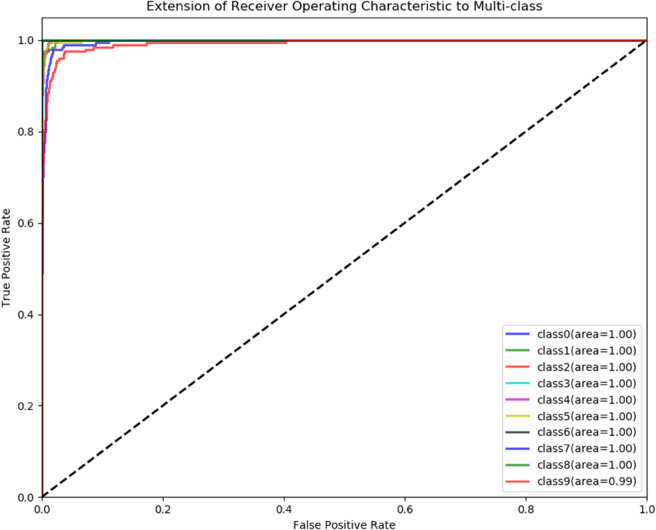
Multi-class discriminability: receiver operating characteristic (ROC) curves for LSTM-L1/2-SCN model with all AUC >0.99 (Case 1).

**Fig 22:**
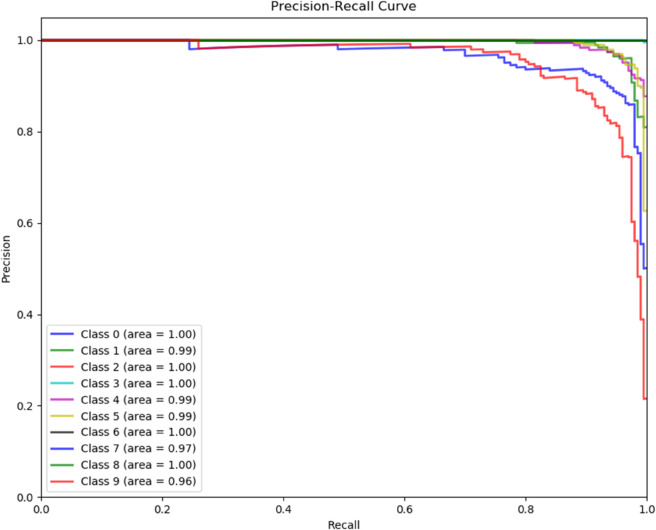
Precision-Recall Dominance: Class-wise curves exhibiting minimum AUPRC of 0.96 with five classes achieving perfection (Case 1).

To demonstrate the training process and sparsity effect of the proposed model, Figs 10 to 13 present the training convergence curves of the model under four cases, while Figs 14 to 17 show the weight distribution on the output side of $L_{1/2}$-SCN. Taking Case 1 as a representative instance, Figs 18 and 19 present the statistical indicators of the proposed model for each type of fault identification and their overall distribution, while Fig 20 presents the confusion matrix based on the test set. Figs 21 and 22 depict the ROC and PR curves, respectively.

To showcase the efficacy of LSTM in feature abstraction and extraction, LSTM-$L_{1/2}$-SCN is compared with $L_{1/2}$-SCN without data feature extraction, with the corresponding fault identification results reported in Table 11. The test accuracy is less than 60%, which infer the important role played by the LSTM model in feature extraction in the first stage.

**Table 11:** Experimental results of $L_{1/2}$-SCN without LSTM.

Case	Training ACC	Test ACC	Sparsity
Case 1	39.62%	35.05%	9.70%
Case 2	63.30%	57.95%	9.68%
Case 3	61.38%	54.25%	10.30%
Case 4	51.44%	45.00%	9.58%

In order to analyze the influence of the value of theegular parameter λ on the sparsity and performance of the model, Table 12 lists the results of LSTM-$L_{1/2}$-SCN when the regular parameters are set to 0.005 and 0.01, respectively. The results infer that the regularization coefficient λ significantly impacts the sparsity of $L_{1/2}$-SCN. The larger λ, the stronger the sparsity. Therefore, choosing the appropriate coefficient requires a parameter-tuning process to balance the two aspects.

**Table 12:** Results of fault identification of rolling bearings with different regularization coefficient.

Case	Training ACC, Test ACC	
	λ=0.005	λ=0.01
Case1	98.46%, 97.20%	98.51%, 97.00%
Case2	98.59%, 97.50%	98.73%, 98.15%
Case3	97.81%, 97.10%	98.43%, 97.30%
Case4	98.17%, 96.80%	98.06%, 96.55%

Table 13 presents the sparsity of LSTM-$L_{1/2}$-SCN across the four working conditions, and Table 14 presents a computational cost comparison between the LSTM-$L_{1/2}$-SCN model and the Attention-TCN-BiLSTM model.

**Table 13:** Sparsity of $L_{1/2}$-SCN.

Case	λ = 0.00	λ = 0.01
Case1	24.24%	33.62%
Case2	23.88%	34.18%
Case3	29.39%	33.82%
Case4	24.72%	34.50%

**Table 14:** Computational cost comparison.

Metrics	LSTM-$L_{1/2}$-SCN	Attention-Tcn-BiLSTM	Ratio
Training time	9.25min	198min	1:21.8
Resource usage	2.5G	6G	1:2.4
FLOPs	74.6G	160.16G	1:2.15
Params	15.9K	157K	1:10

To further verify the generalization performance of the model, Table 15 validates the performance of the proposed model based on noisy datasets (with noise added).

**Table 15:** Comparison of LSTM-$L_{1/2}$-SCN performance before and after adding noise to the dataset (Case 1).

Model	Evaluation indexes (%, Mean ± 95% CI)				
	Test ACC	Precision	Recall	Macro F1	Macro AUC
Before adding noise	**0.9728±0.0096**	**0.9728±0.0096**	**0.9727±0.0096**	**0.9725±0.0100**	**0.9989±0.0003**
After adding noise	0.9706±0.0008	0.9710±0.0009	0.9706±0.0008	0.9705±0.0009	0.9197±0.0022

To highlight the overall merits of the proposed method, Table 16 compares its performance with other models in terms of sparsity and classification accuracy. The values reported represent the average experimental results across four operating conditions derived from the CWRU dataset, whereas the accuracy of competing models is averaged based on their suboptimal experimental outcomes.

**Table 16:** Comparison and summary of LSTM-$L_{1/2}$-SCN and others.

	LSTM-$L_{1/2}$-SCN	Others
Sparsity	25.56%	N/A
Accuracy	97.17%	96.69%

### Results analysis and discussion

#### Benchmark experiments.

The results on five benchmark datasets (Tables 3 and Table 4, Figs 4 to 7) demonstrate that $L_{1/2}$-SCN exhibits superior sparsity and generalization capabilities. Regarding sparsity performance, when compared to PSCN, $L_{1/2}$-SCN has a maximum increase of 56%. This is primarily due to integrating $L_{1/2}$ regularization into SCN, which offers better sparsity than the *L*_1_ regularization. It also results in a significant number of zero weights, effectively preventing overfitting and enhancing the generalization ability.

#### Fault diagnosis experiments.

Statistics metrics analysis: Tables 6 to 9 show that LSTM-$L_{1/2}$-SCN performs exceptionally well on the mean values of all five indicators. Take condition 1 as an example. The Test ACC of LSTM-$L_{1/2}$-SCNis 97.28%, which is 0.22 percentage points higher than that of the suboptimal model Attention-TCN-BiLSTM. The Precision is 0.9729, which is 0.21 percentage points higher than that of the suboptimal model. The Recall is 0.9728, which is 0.21 percentage points higher than that of the suboptimal model. F1 is 0.9725, which is 0.17 percentage points higher than that of the suboptimal model. The AUC is 0.9989, which is 6.41 percentage points higher than that of the suboptimal model. These indicators illustrate the superiority of the model in terms of accuracy. However, the drawback of LSTM-$L_{1/2}$-SCN is that the variance of the experimental results is relatively large, which indicates that the randomness of the model parameter values is still relatively high and requires subsequent improvement. Figs 10 to 13 show the training convergence curves of the proposed model for one experiment conducted under each of the four working conditions. It can be seen that when the number of hidden layer nodes increases to 500, the accuracy rate of the model on the training set can all exceed 98%, indicating the good performance of the model. Figs 14 to 17 present the weight distribution of $L_{1/2}$-SCN in the fusion model, the percentage of zero weight is above 23%, verifying the sparse effect.

Confusion matrix analysis: In order to observe the intuitive recognition of various types of faults by the model, Fig 20 presents the confusion matrix of the test set in a certain experiment. It can be seen that the classification effect of the model for categories 8 and 10 is not good. These two categories are ’a 0.014-inch fault on the bearing outer ring at 6 o’clock’ and ’Normal state’. This is related to the data distribution and quality to some extent. As can be seen from Fig 8, the vibration periodicity of the 8th type of data is poor and the data variance is large, while the 10th type of data is affected by some outliers (noise). This will affect the learning effect of the model and thereby the classification effect.

The paired t-test analysis (Table 10): The results of the paired t-test reveal statistically significant disparities between the proposed model and the a-cnn-bilstm model across five pivotal performance metrics: test accuracy (test acc), precision, recall, macro F1 score, and macro AUC. Specifically, for all five metrics, the t-statistics exhibit relatively high values, with corresponding p-values substantially below the conventional significance threshold of 0.05. This finding underscores that the observed performance differences between the two models are unlikely to be attributable to random variation and are instead statistically robust. Notably, in the macro AUC metric, the t-statistic reaches an exceptionally high value of 79.0984, accompanied by a p-value approaching zero. This compelling evidence further substantiates that the proposed model outperforms the a-cnn-bilstm model markedly in discriminating between positive and negative samples.

ROC and PR curve analysis (Figs 21 and 22): The ROC curves closely approach the top-left corner, with a minimum AUC value of 0.98 and an average AUC exceeding 0.99 across all samples, indicating high classification accuracy across all thresholds. As evidenced by a well-behaved ROC curve indicating stable model performance. Similarly, the PR curves, boast a minimum of 0.91 and an average of 0.987, highlighting the model’s balance between accuracy and recall rates, underscoring its better performance.

Analysis of the Feature Extraction Function of LSTM (Table 11): The first stage uses only the basic LSTM model to maintain model simplicity, avoiding more complex alternatives like BiLSTM. A comparative experiment underscores LSTM’s role in feature extraction, contrasting the $L_{1/2}$-SCN model without LSTM. In Table 11, the average test ACC of $L_{1/2}$-SCN without LSTM under the same parameter settings is 48.06%. Therefore, the shallow model alone exhibits limitations in handling large-scale data, with suboptimal fault identification performance using solely $L_{1/2}$-SCN. Hence, fusing LSTM and $L_{1/2}$-SCN can better realize fault identification.

Sparsity and the regularization parameter λ analysis (Tables 12 and 13): Table 13 highlights that the sparsity of $L_{1/2}$-SCN surpasses 23% when the regularization coefficient λ is set to 0.005, and it can even exceed 33% when the coefficient increases to 0.01. This indicates that SCN attains better sparsity when enhanced with the $L_{1/2}$ regularization technique. Although a larger regularization coefficient generally leads to better sparsity, striking a balance is crucial, as excessively sparse models can result in reduced effective weights, ultimately compromising the model’s accuracy. Therefore, we need to select an appropriate value for λ. Based on the results of the sensitivity experiment in this paper, λ is selected as 0.005. Figs 14 to 17 illustrate the weight distribution, revealing that the zero weights are almost uniformly generated during the gradual increase of the model’s hidden units, which is determined by the principle of $L_{1/2}$ regularization and is also in line with our expectations. In summary, $L_{1/2}$-SCN is used for fault identification in the second stage, affording better sparsity. On the test set, this model achieves an accuracy of 97.20% while maintaining a sparsity level exceeding 23%. Compared to current deep learning models, such as TCN and LSTM, the proposed approach exhibits distinct advantages in terms of sparsity, proving the validity of the $L_{1/2}$-SCN fusion sparse algorithm.

Computational Cost (Table 14): Table 14 reveals that the LSTM-$L_{1/2}$-SCN model demonstrates a notable computational efficiency advantage, with training time reduced to 1/21.8 of the attention mechanism-based temporal model (9.25 minutes per training session), peak memory usage controlled at 2.5GB (58% lower than the 6GB of the comparative model), floating-point operations decreased by 53%, and parameter size only 10% of the comparative model; this efficiency stems from triple optimization—LSTM sequence modeling avoiding large convolution kernel calculations, $L_{1/2}$ regularization eliminating redundant connections via sparse constraints, and an incremental node growth mechanism dynamically adjusting network complexity—making it suitable for deployment in edge computing units of resource-constrained industrial equipment.

Generalization Ability Analysis (Table 15): To verify the robustness and generalization performance of the model, Gaussian noise with a zero - mean and a standard deviation of 0.05, as well as uniformly distributed perturbations with an amplitude range of [-0.05, 0.05], were added, increasing the diversity of the data set. Table 15 presents the various statistical indicators for the model’s classification of the new data set. It can be seen that when the model processes the data after adding noise, in terms of Test ACC, Precision, Recall, and F1, the indicators have decreased on average by approximately 0.2 percentage points, and in terms of AUC, they have decreased by 9 percentage points. However, even when exposed to noise, the model can still maintain a level of more than 90% in key performance indicators. This indicates that when facing a certain degree of data change, the core classification and prediction capabilities of the model have not been fundamentally damaged.

In conclusion, the enhanced performance of the proposed method stems primarily from its unique model structure, differing from conventional deep models. The seven deep learning models in the comparison perform end-to-end tasks, integrating two steps into one process. However, their feature mapping lacks a theoretical foundation. On the contrary, the proposed approach employs LSTM as a feature extractor, effectively condensing the original data while preserving historical time information. This compressed data is input into a shallow model, SCN, leveraging its universal approximation capability. We further refine SCN’s structure with $L_{1/2}$ regularization, enhancing conciseness and minimizing redundancy. This two-stage learning model exhibits sparsity and achieves higher accuracy, leading to improved fault identification results.

## Conclusion

This study presents an integrated LSTM and $L_{1/2}$-SCN architecture for rolling bearing fault diagnosis. By fusing temporal feature extraction with non-convex sparse regularization, the model achieves 25.56% weight sparsity (achieves an average improvement of 24.8% over PSCN) while reducing training duration by 95.3%. Convergence is guaranteed through a reconstructed supervision mechanism validated by mathematical formulas. Testing on the CWRU 10-class dataset yields 97.17% accuracy - surpassing comparable deep models by 0.2-10 percentage points. The implementation demonstrates industrial viability by enabling real-time diagnosis, which is suitable for edge deployment in rotating machinery monitoring systems.

Nevertheless, the model exhibits limitations under extreme variable operating conditions, particularly in multi-fault coupling scenarios. These constraints originate from the inherent non-stationarity of vibration signals and the current feature extraction mechanism’s limited frequency band adaptability.

Future work will focus on exploring the comprehensive integration of multimodal information to further enhance the modeling and prediction capabilities in complex scenarios. Specifically, the idea of integrating multi-scale time series modules for prediction [33] and the relational interaction modeling method [34] can be applied. Meanwhile, this work will explore the architectural design of a modal fusion Vision Transformer (ViT), similar to [35], and the multimodal deep learning scheme outlined in [36], and study the fusion strategies for lightweight and adaptive models. This direction aims to build a more flexible multimodal fusion system to solve complex problems involving multi-source heterogeneous data. To ensure the practical deployment of such advanced systems, future work will also involve benchmarking the models on specific edge platforms and evaluating key metrics such as inference latency and power consumption.
